# Lymphangioleiomyomatosis Biomarkers Linked to Lung Metastatic Potential and Cell Stemness

**DOI:** 10.1371/journal.pone.0132546

**Published:** 2015-07-13

**Authors:** Gorka Ruiz de Garibay, Carmen Herranz, Alicia Llorente, Jacopo Boni, Jordi Serra-Musach, Francesca Mateo, Helena Aguilar, Laia Gómez-Baldó, Anna Petit, August Vidal, Fina Climent, Javier Hernández-Losa, Álex Cordero, Eva González-Suárez, José Vicente Sánchez-Mut, Manel Esteller, Roger Llatjós, Mar Varela, José Ignacio López, Nadia García, Ana I. Extremera, Anna Gumà, Raúl Ortega, María Jesús Plà, Adela Fernández, Sònia Pernas, Catalina Falo, Idoia Morilla, Miriam Campos, Miguel Gil, Antonio Román, María Molina-Molina, Piedad Ussetti, Rosalía Laporta, Claudia Valenzuela, Julio Ancochea, Antoni Xaubet, Álvaro Casanova, Miguel Angel Pujana

**Affiliations:** 1 Program Against Cancer Therapeutic Resistance (ProCURE), Breast Cancer and Systems Biology, Catalan Institute of Oncology (ICO), Bellvitge Institute for Biomedical Research (IDIBELL), L’Hospitalet del Llobregat, Catalonia, Spain; 2 Department of Pathology, University Hospital of Bellvitge, Bellvitge Institute for Biomedical Research (IDIBELL), L’Hospitalet del Llobregat, Catalonia, Spain; 3 Department of Pathology, Vall d'Hebron Hospital, Barcelona, Catalonia, Spain; 4 Cancer Epigenetics and Biology Program, Bellvitge Institute for Biomedical Research (IDIBELL), L’Hospitalet del Llobregat, Catalonia, Spain; 5 Department of Physiological Sciences II, School of Medicine, University of Barcelona, Barcelona, Catalonia, Spain; 6 Institució Catalana de Recerca i Estudis Avançats (ICREA), Barcelona, Catalonia, Spain; 7 Cruces University Hospital, BioCruces Research Institute, University of the Basque Country, Barakaldo, Spain; 8 Department of Radiology, University Hospital of Bellvitge, Bellvitge Institute for Biomedical Research (IDIBELL), L’Hospitalet del Llobregat, Catalonia, Spain; 9 Department of Gynecology, University Hospital of Bellvitge, Breast Cancer Unit, Catalan Institute of Oncology (ICO), Bellvitge Institute for Biomedical Research (IDIBELL), L’Hospitalet del Llobregat, Catalonia, Spain; 10 Department of Medical Oncology, Breast Cancer Unit, Catalan Institute of Oncology (ICO), Bellvitge Institute for Biomedical Research (IDIBELL), L’Hospitalet del Llobregat, Catalonia, Spain; 11 Department of Pulmonology, Lung Transplant Unit, Lymphangioleiomyomatosis (LAM) Clinic, Vall d'Hebron University Hospital, Barcelona, Catalonia, Spain; 12 Department of Pneumology, University Hospital of Bellvitge, Bellvitge Institute for Biomedical Research (IDIBELL), L’Hospitalet del Llobregat, Catalonia, Spain; 13 Biomedical Research Centre Network for Respiratory Diseases (CIBERES), Madrid, Spain; 14 Department of Pneumology, University Hospital Clínica Puerta del Hierro, Madrid, Spain; 15 Department of Pneumology, Instituto de Investigación Sanitaria La Princesa, Hospital La Princesa, Madrid, Spain; 16 Department of Pneumology, Hospital Clinic of Barcelona, Agusti Pi Suñer Biomedical Research Institute (IDIBAPS), Barcelona, Catalonia, Spain; 17 Department of Pneumology, Henares Hospital, Madrid, Spain; Texas A&M University Health Science Center, UNITED STATES

## Abstract

Lymphangioleiomyomatosis (LAM) is a rare lung-metastasizing neoplasm caused by the proliferation of smooth muscle-like cells that commonly carry loss-of-function mutations in either the tuberous sclerosis complex 1 or 2 (*TSC1* or *TSC2*) genes. While allosteric inhibition of the mechanistic target of rapamycin (mTOR) has shown substantial clinical benefit, complementary therapies are required to improve response and/or to treat specific patients. However, there is a lack of LAM biomarkers that could potentially be used to monitor the disease and to develop other targeted therapies. We hypothesized that the mediators of cancer metastasis to lung, particularly in breast cancer, also play a relevant role in LAM. Analyses across independent breast cancer datasets revealed associations between low *TSC1/2* expression, altered mTOR complex 1 (mTORC1) pathway signaling, and metastasis to lung. Subsequently, immunohistochemical analyses of 23 LAM lesions revealed positivity in all cases for the lung metastasis mediators fascin 1 (FSCN1) and inhibitor of DNA binding 1 (ID1). Moreover, assessment of breast cancer stem or luminal progenitor cell biomarkers showed positivity in most LAM tissue for the aldehyde dehydrogenase 1 (ALDH1), integrin-ß3 (ITGB3/CD61), and/or the sex-determining region Y-box 9 (SOX9) proteins. The immunohistochemical analyses also provided evidence of heterogeneity between and within LAM cases. The analysis of *Tsc2*-deficient cells revealed relative over-expression of FSCN1 and ID1; however, *Tsc2*-deficient cells did not show higher sensitivity to ID1-based cancer inhibitors. Collectively, the results of this study reveal novel LAM biomarkers linked to breast cancer metastasis to lung and to cell stemness, which in turn might guide the assessment of additional or complementary therapeutic opportunities for LAM.

## Introduction

LAM is a rare lung disease that appears predominantly in women of childbearing age and is depicted by cystic lung destruction [1–4]. LAM results from the proliferation of typically estrogen receptor alpha (ERa)- and progesterone receptor (PR)-positive smooth muscle-like cells [5–7] with lung metastatic potential [8,9]. Importantly, LAM cells commonly carry loss-of-function mutations in either the *TSC1* or *TSC2* tumor suppressor gene [10–13] and, consequently, exhibit abnormal activation of mTORC1 [14,15]. Thus, allosteric mTOR inhibition has demonstrated substantial clinical benefit in LAM patients [16]; however, complementary therapies are still required to improve the response and/or to treat specific patients [16,17].

Intriguingly, diverse data indicate that LAM cells originate from a different organ to the lung [9]; for example, LAM cells can be found circulating in the blood and lymphatic systems [18,19], and LAM lesions can reappear after lung transplantation, although not derived from the tissue donor [20,21]. Thus, a specific cell type(s) may possess metastatic potential with lung tropism when, most commonly, a *TSC1* or *TSC2* mutation is acquired and mTORC1 is abnormally activated. Interestingly, however, there are recorded cases of sporadic LAM without mutations in *TSC1* or *TSC2* and, therefore, without abnormal activation of mTORC1 [22].

mTORC1 regulates a cancer invasion and metastatic transcriptional program [23]. Notably, in breast cancer, relative low expression of hamartin and tuberin (*TSC1* and *TSC2* products, respectively) is associated with poor clinical outcome [24], and depletion of tuberin promotes metastasis [25]. Together, these observations led us to hypothesize that, beyond the proposed role for chemokines [26], the mediators of breast cancer metastasis to lung could also play a role in LAM. To assess this hypothesis, we analyzed breast cancer gene expression data and subsequently evaluated the presence of defined metastasis mediators in LAM tissue.

## Materials and Methods

### Breast cancer gene expression analyses

Data for gene expression profiles of metastatic breast cancer were taken from the corresponding publication [27] and from the Gene Expression Omnibus (GEO) references GSE11078 [28] and GSE2034 [29]. The osteosarcoma dataset was downloaded from GSE14827 [30]. Pre-processed and normalized gene expression data were downloaded from the corresponding repository of The Cancer Genome Atlas (TCGA) (July 3, 2012; http://tcga-data.nci.nih.gov/tcga/tcgaHome2.jsp). Expression profiles were clustered based on the PAM50 signature, which assigns tumors to the basal-like, HER2-enriched, luminal A or luminal B breast cancer subtypes. The MCF7 RNAs were extracted using TRIzol (Life Technologies) and, following quality control and labeling, hybridized on the Human Genome U133 Plus 2.0 Array microarray platform (Affymetrix). The RMA method implemented in BioConductor was applied for background adjustment and normalization of intensity values. The data have been deposited under the GEO reference GSE68324. The Gene Set Expression Analysis (GSEA) [31] tool was run using default values for all parameters. The GSEA analyses used pathway annotations from the Kyoto Encyclopedia of Genes and Genomes (KEGG) [32].

### shRNA assays

The short-hairpin (sh) RNA sequence targeting the expression of *TSC2* corresponded to a construct from the MISSION (Sigma-Aldrich) library and has been described previously [33,34]. The lentiviral packaging, envelope, control and GFP expression plasmids (psPAX2, pMD2.G, non-hairpin-pLKO.1, scrambled-pLKO.1, and pWPT-GFP, respectively) were purchased from Addgene. Production and collection of lentiviral particles followed a modified Addgene protocol. Initial viral titers > 5 x 10^5^/ml were confirmed by Lenti-X GoStix (Clontech) and supernatants were then concentrated by ultracentrifugation or Lenti-X Concentrator (Clontech) and stored at −80°C. Concentrated viral supernatants were titrated for optimal inhibition of the target.

### LAM patients

LAM patients were recruited and lung tissue samples collected by the centers participating in this study and with the support of the Spanish LAM Association (AELAM). Part of this cohort has been described previously [35]. All patients provided written informed consent and the study was approved by the ethics committees of the Bellvitge Institute for Biomedical Research (IDIBELL) and the Instituto de Investigación Sanitaria La Princesa (SEPAR-2012). LAM clinical data included year of birth, year of first symptom, all symptoms presented (included pneumothorax and chylothorax), lung transplantation, presence of angiomyolipomas and/or uterine myomas, treatment, smoker status (previous and current), and comorbidity with other diseases (S1 Table).

### Antibodies

The antibodies used in this study were anti-ALDH1 (catalog #611194, BD Biosciences), anti-CD61 (#EP2417Y, Novus), anti-ERa (#IR151, Dako), anti-FSCN1 (#SC-56531, Santa Cruz Biotechnology), anti-premelanosome protein (anti-HMB-45; #SC-59305, Santa Cruz Biotechnology), anti-ID1 (#SC-488, Santa Cruz Biotechnology), anti-PR (#IR168, Dako), anti-phospho-Ser235-236 S6 ribosomal protein (anti-pS6; clone 91B2, Cell Signaling Technology), anti-actin alpha-smooth muscle (anti-SMA; #A2547, Sigma-Aldrich), anti-SOX9 (#AB5535, Millipore), and anti-tubulin alpha (TUBA; clone DM1A+DM1B, Abcam). Additional proteins/antibodies were evaluated, but the corresponding immunohistochemistry results were not conclusive of specific signals; they corresponded to anti-epiregulin (anti-EREG; #AF1195, RD Systems), anti-keratin 81 (anti-KRT81; #NBP1-69809, Novus Biologicals), anti-retinoic acid receptor responder 3 (anti-RARRES3; #HPA011219, Sigma-Aldrich), and anti-vascular cell adhesion molecule 1 (anti-VCAM1; #551147, BD Biosciences).

### Immunohistochemistry

Immunohistochemical assays were performed using standard protocols with the EnVision (Dako) method. Each tissue and biomarker was evaluated in at least two independent assays and no substantial differences were observed. Equivalent sections were processed to include incubation with immunoglobulin controls (Sigma-Aldrich), which did not reveal staining in any case. Secondary antibodies for immunofluorescence (Alexa) were obtained from Molecular Probes (Life Technologies). The immunohistochemistry results were scored independently and blindly (to molecular and clinical status) by two expert pathologists. The association between biomarkers was assessed by computing the Spearman’s correlation coefficient (SCC).

### Cell culture

The cell lines were cultured following standard protocols and cellular viability was evaluated by performing methylthiazol tetrazolium (MTT, Sigma-Aldrich) assays. Everolimus was purchased from Selleck Chemicals and the inhibitors of ID1 expression or stability (apigenin, C527, and cannabidiol) from Sigma-Aldrich. The results correspond to two independent assays for each compound and to triplicates for each data point. Given the half-maximal inhibitory concentration (IC_50_) and the maximal response to everolimus in *Tsc2* ^-/-^/*Tp53* ^-/-^ murine embryonic fibroblasts (MEFs) and *Tsc2*-deficient Eker rat leiomyoma (ELT3) cells, these cells were exposed to 1 and 100 μM of the rapalog, respectively.

## Results

### Low *TSC1/2* expression in breast tumors that metastasize to the lung

**Fig 1:**
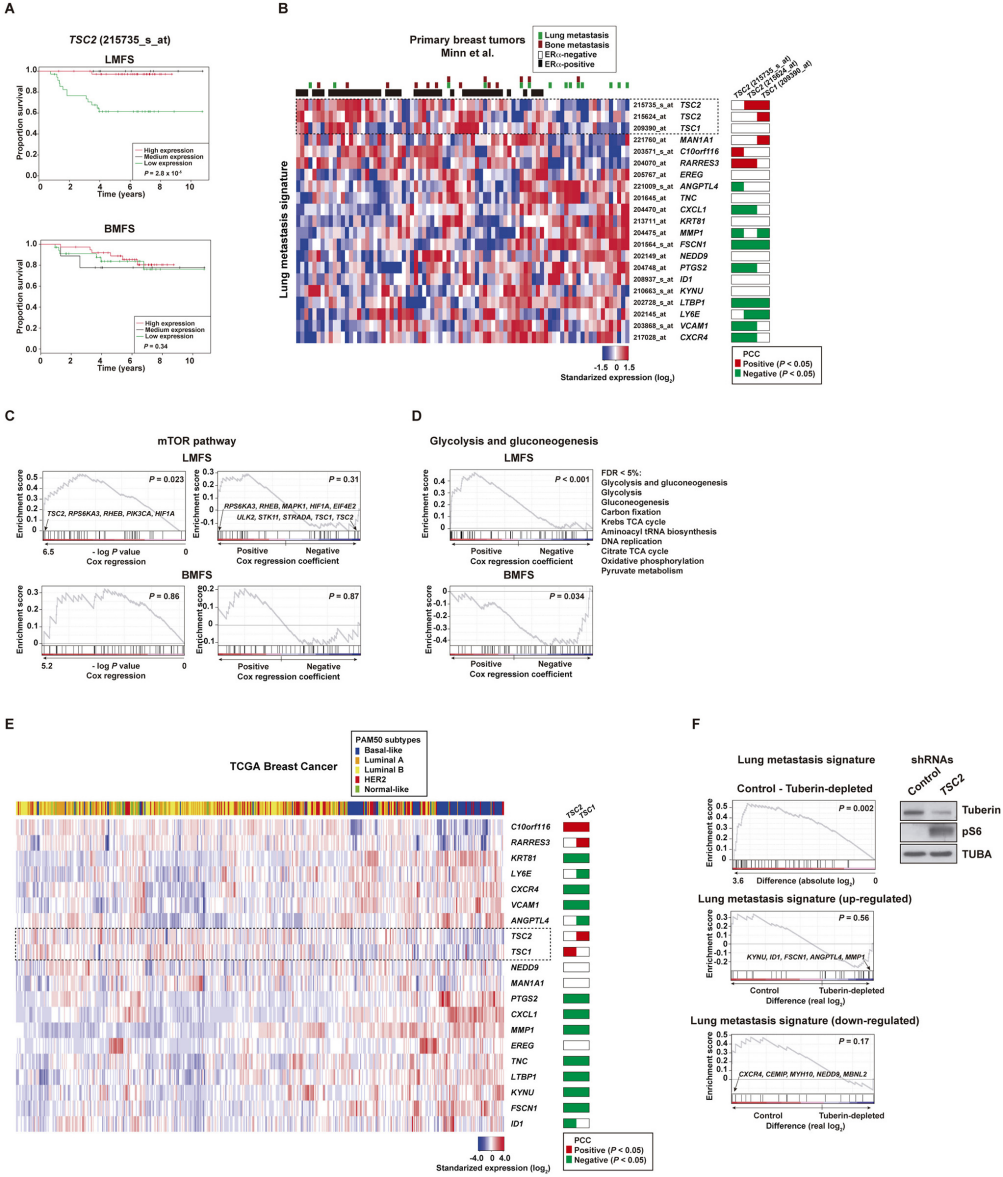
Expression of mTOR pathway components and breast cancer metastasis to lung. (A) Kaplan-Meier lung metastasis-free survival (LMFS) and bone metastasis-free survival (BMFS) curves based on categorization of *TSC2* expression. The *P* values of the Cox proportional-hazards regression analysis are shown. (B) Tumor sample and gene expression clustering, and correlations of *TSC1/2* and genes from the lung metastasis signature, in the seminal breast cancer dataset [27]. (C) GSEA results for Cox regression values of the mTOR pathway gene set and LMFS or BMFS. (D) GSEA results for Cox regression values of metabolic pathway gene sets and LMFS or BMFS. (E) Tumor sample and gene expression clustering, and correlations between *TSC1/2* and genes from lung metastasis signature, in the TCGA dataset [70]. (F) GSEA results for the expression difference of the lung metastasis signature between MCF7 cells transduced with control or *TSC2*-target shRNAs. The left top panel shows the results for absolute expression differences, and the middle and bottom panels show the results for real differences of the up-regulated and down-regulated subsets of the signature, respectively. The right panels show the Western blot results for tuberin, pS6 and control loading, TUBA.

The link between *TSC1/2* expression, mTORC1 activity and breast cancer metastatic potential was primarily investigated by analyzing publicly available gene expression datasets. Relatively low *TSC2* expression was found to be significantly associated with lung but not bone metastasis events in the analysis of a seminal breast cancer dataset [27] (Fig 1A). Consistent with clinical observations [36], lung metastatic events were preferentially linked to ERa-negative tumors (Fig 1B) and the above association was also significant in this subtype (*P* = 0.029). Thus, low expression of *TSC2* (and of *TSC1*, although the univariate association was not significant: *P* = 0.09) was evident in the tumors that caused lung metastases (Fig 1B). Consequently, *TSC1* and/or *TSC2* were found to be significantly co-expressed (Pearson’s correlation coefficient (PCC) *P* values < 0.05) in the expected direction of mediating lung metastasis with 10 of the 18 the genes that made up the seminal lung metastasis signature [27] (positively correlated: *C10orf116*, *MAN1A1*, and *RARRES3*; and negatively correlated: *ANGPTL4*, *CXCL1*, *FSCN1*, *LTBP1*, *MMP1*, *PTGS2*, and *VCAM1*; *CXCR4* and *LY6E* were correlated in the opposite expected direction; Fig 1B).

Beyond the specific analysis of *TSC1/2*, a pathway-based analysis of the same breast cancer dataset suggested an association between mTORC1 activity and lung but not bone metastasis events (Fig 1C). In this analysis, suppressors and activators of the mTORC1 pathway pointed to increased signaling linked to lung metastatic potential (Fig 1C). Moreover, consistent with the role of mTOR as a nutrient sensor, significant associations were also found between metabolic pathways and lung but not bone metastasis (Fig 1D).

Next, analysis of an independent dataset from primary breast tumors [28] also showed low *TSC1* and *TSC2* expression to be specifically associated with lung metastasis (S1A Fig). The same dataset also revealed an analogous association with differential expression of genes from the mTOR pathway (S1B Fig). Moreover, analysis of a third dataset [29] confirmed the association between relatively low *TSC2* expression and poor survival of patients with ERα-negative tumors (S1C Fig). In addition, analysis of the TCGA breast cancer dataset revealed significant under-expression of *TSC1/2* in primary tumors that are expected to preferentially metastasize to lung (basal-like type [36]; two-tailed *t*-test *P* values < 0.001) and, consistently with this observation, *TSC1/2* were found to be co-expressed with most of the genes that constitute the seminal lung metastasis signature [27]: 13 of the 18 signature genes showed significant PCCs in the expected direction of mediating lung metastasis (positively correlated: *C10orf116* and *RARRES3*; and negatively correlated: *ANGPTL4*, *CXCL1*, *FSCN1*, *ID1*, *KYNU*, *KRT81/KRTHB1*, *LTBP1*, *MMP1*, *PTGS2*, *TNC*, and *VCAM1*; Fig 1E). Lastly, in support of the above observations, depletion of tuberin expression by a short-hairpin RNA transduced into breast cancer cells with wild-type *TSC2*, MCF7, revealed a significant change in the expression of the lung metastasis signature (*P* = 0.002); thus, genes that when up-regulated mediate lung metastasis showed a trend to be up-regulated with tuberin depletion, and the opposite trend was observed for the down-regulated set (Fig 1F).

Remarkably, although the lung is the most common metastatic site for other cancer types, in particular for osteosarcoma, analysis of an equivalent microarray dataset [30] did not reveal similar associations (S2 Fig). Collectively, these results suggest a specific link between loss of *TSC1/2* expression—and probably, therefore, activated mTORC1 signaling—and lung metastatic potential in breast cancer.

### Biomarkers of lung metastatic potential in LAM lesions

**Fig 2:**
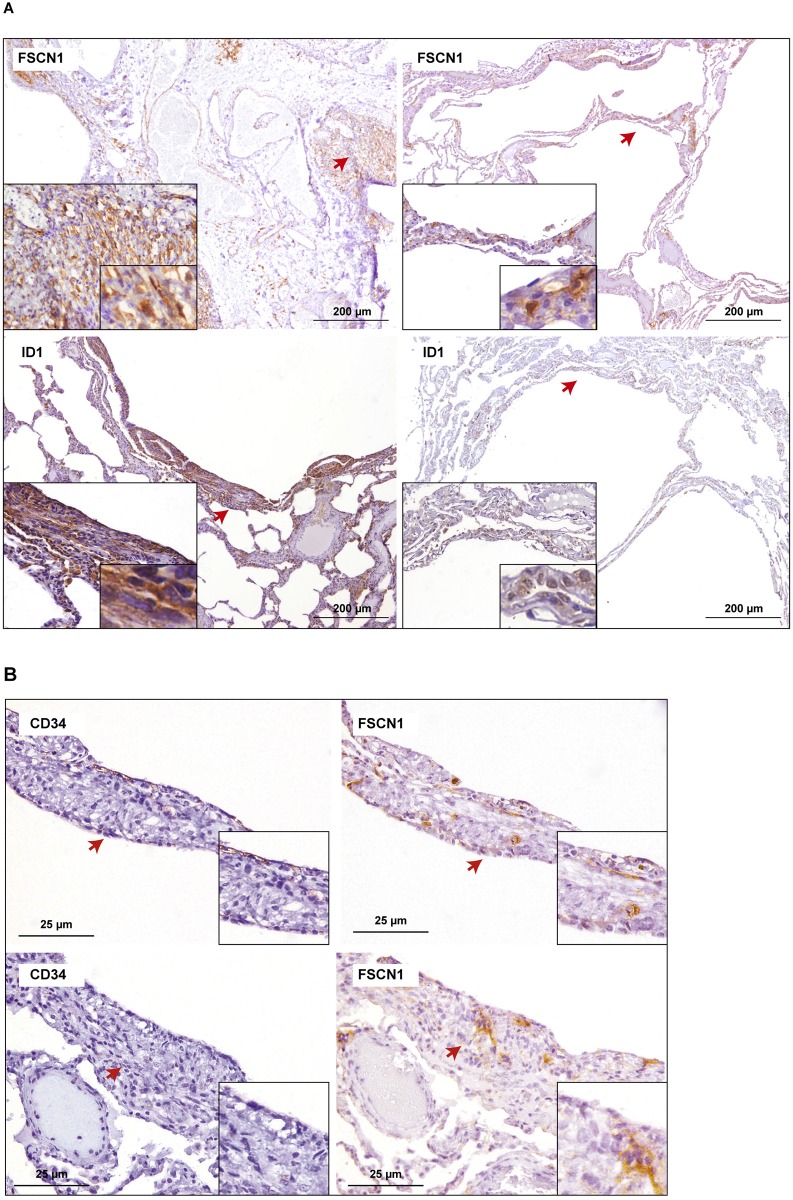
Positivity for breast cancer lung metastasis mediators in LAM tissue. (A) Representative results for FSCN1 and ID1 in two LAM cases. Arrows mark magnified fields shown in the insets. (B) Representative immunohistochemistry results for CD34 and FSCN1 in LAM tissue. Positivity for FSCN1 is greater and not limited to endothelial cells.

**Fig 3:**
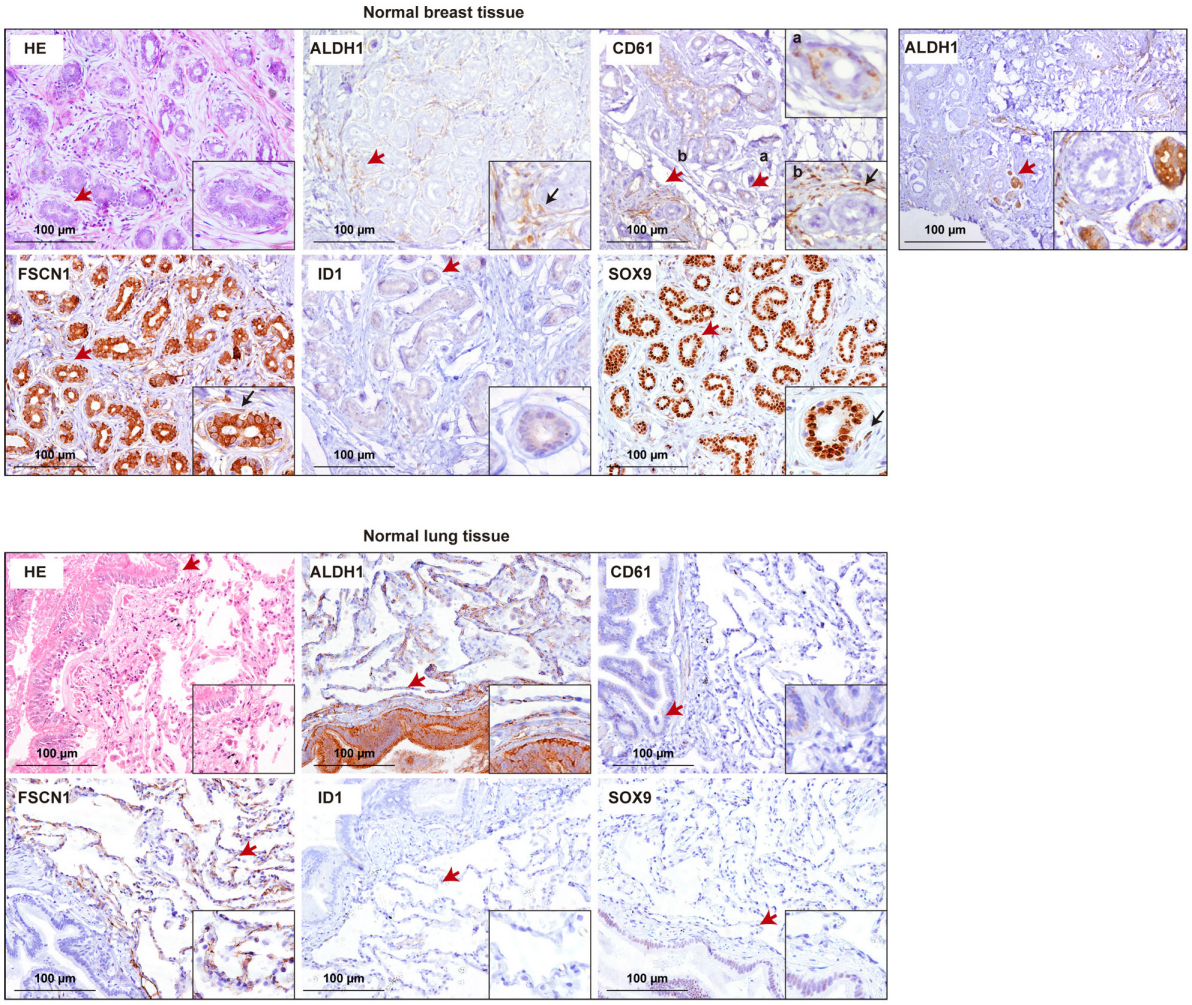
Immunohistochemical characterization of biomarkers in normal breast and lung tissue. (A) Representative hematoxylin-eosin and immunohistochemical staining in normal breast tissue. The observed patterns of positivity were those expected with the exception of ALDH1, which could have showed positivity in the basal and luminal cell layers of the acini; nonetheless, this can only be observed at the growing end and branching of the ducts [48], which may be represented by the image shown in the right panel. Expression of ALDH1, CD61, FSCN1 and SOX9 was also seen in spindle-like cells surrounding the terminal extra-lobular ducts as well as in similar cells of the loose specialized intra-lobular stroma (arrows in insets). The results of CD61 are detailed for the basal cell layer in differentiated acini (a) and for spindle-like intra-lobular cells (b). The arrows mark magnified fields. (B) Representative hematoxylin-eosin and immunohistochemical staining in normal lung tissue. ALDH1 and FSCN1 mark the alveolar endothelium, and ALDH1 also marks the basal and luminal layers of the bronchioles. CD61, ID1 and SOX9 are not expressed in differentiated alveoli, and CD61 and SOX9 show positivity in the luminal and/or basal layers of the bronchioles.

Having suggested an association between *TSC1/2* expression and the lung metastatic potential of breast cancer, we performed immunohistochemistry studies of the lung metastasis mediators in paraffin-embedded lung tissue from 23 LAM patients. Hematoxylin-eosin staining of all LAM lung tissue revealed the characteristic cystic abnormalities (S3A Fig), and immunohistochemical analyses showed some degree of positivity for common diagnostic markers (S3B Fig). Next, several antibodies that may recognize proteins encoded by the breast cancer lung metastasis signature [27] were assessed by immunohistochemical assays. Positivity in LAM lesions was revealed for FSCN1 and ID1 (Fig 2A). FSCN1 is an endothelial cell biomarker that also identifies differentiated luminal and spindle-like cells in normal breast tissue (Fig 3), and which has been extensively linked to the metastatic potential of cancer cells [37]. Notably, the staining pattern of FSCN1 in LAM lesions was found to be more extensive than that of a normal endothelial biomarker, CD34 (Fig 2B). In addition, all cases showed positivity for both metastatic biomarkers (S1 Table) and their intensity scores were somewhat correlated (SCC = 0.38, one-tailed *P* = 0.041).

The subcellular localization of ID1 was prominently cytoplasmic in most cases (Fig 2A, bottom left panel), although major nuclear localization was also observed (Fig 2A, bottom right panel). Notably, cytoplasmic-nuclear shuttling of ID proteins regulates their function and cytoplasmic ID1 has been implicated in active angiogenesis [38]. Examination of normal breast and lung tissue for ID1 expression revealed weak positivity (in luminal cells) and negativity, respectively (Fig 3). Together, this study reveals the presence of two biomarkers (defined as linked to a specific biological function) of lung metastatic potential in LAM lesions.

### Cell stemness biomarkers in LAM lesions

mTORC1 regulates hematopoietic stem cell homeostasis and FSCN1 is a key mediator of this function [39]. In addition, the ID proteins regulate stem cell phenotypes [40] and, particularly, ID1 maintains embryonic stem cell self-renewal [41]. In breast cancer, over-expression of canonical stem and/or progenitor cell biomarkers, such as ALDH1 and CD61, has been associated with poor-prognosis [42,43]. CD61 defines a mammary cell population postulated to contribute to the origin of the tumor subtype that preferentially metastasizes to lung [44]. In this setting, SOX9 has a key role conferring lung metastasis-seeding properties and, intriguingly, has also been identified as a lung stem cell biomarker [45–47]. In normal breast tissue, ALDH1, CD61 and SOX9 (and FSCN1) also mark spindle-like cells within the loose specialized intra-lobular stroma and/or surrounding the terminal extra-lobular ducts (Fig 3), which is consistent with previous observations for other stem/progenitor cell markers of mammary development [48].

**Fig 4:**
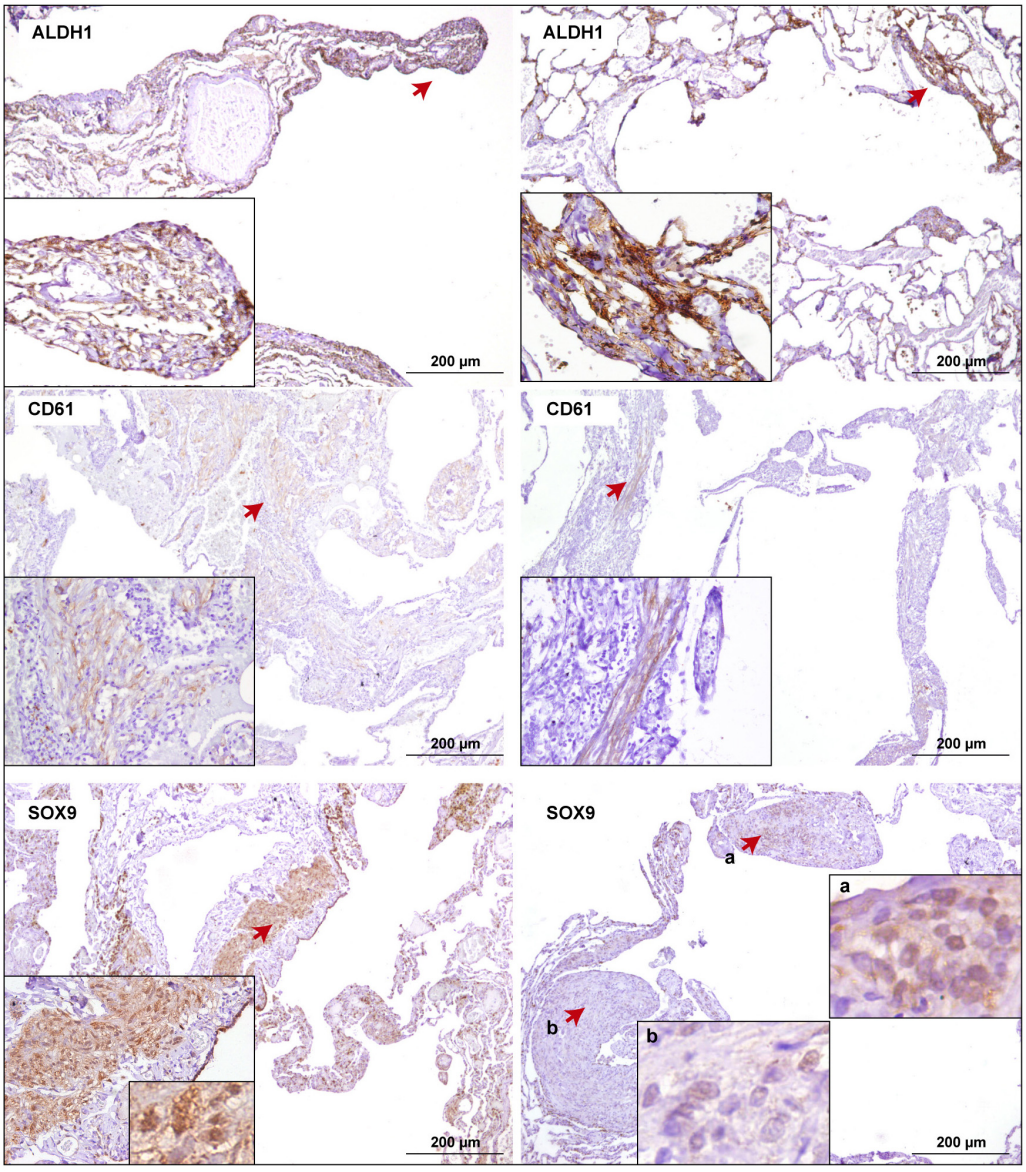
Positivity for breast cancer stemness biomarkers in LAM tissue. Representative positive results for ALDH1, CD61 and SOX9 in two LAM cases. Arrows mark magnified fields shown in the insets.

The above observations led us to assess the expression of ALDH1, CD61 and SOX9 in the LAM lesions. The results showed positivity in 90%, 63%, and 77% of the cases, respectively (Fig 4 and S1 Table). There were no significant correlations between the expression of these and/or the above biomarkers; nevertheless, CD61 showed a trend for positive correlations (SCCs > 0.20) with FSCN1, ID1 and SOX9, but a negative correlation (SCCs < -0.20) with ALDH1. Studies in larger tissue series may be required to corroborate these trends.

Analogous to the observations for ID1, SOX9 showed heterogeneity between cytoplasmic and nuclear sub-cellular staining (Fig 4, SOX9 left and right panels, respectively); however, normal differentiated lung was found to be mostly negative for this biomarker (Fig 3). Considering the potential influence of SOX9 in LAM pathogenesis, cytoplasmic positivity has been associated with invasive breast cancer and metastasis, but normal differentiated mammary epithelia generally exhibit nuclear localization (Fig 3) [45]. In addition, the staining of SOX9 was also found to be heterogeneous intra-LAM tissue (Fig 4, right panels). Notably, in normal breast tissue, pS6 and SMA staining identifies different cell populations, including the basal layer of ducts [49,50]. HMB-45 is generally negative in this tissue, but positivity has been observed in breast tumors with a myoepithelial or melanocytic phenotype [51,52].

**Fig 5:**
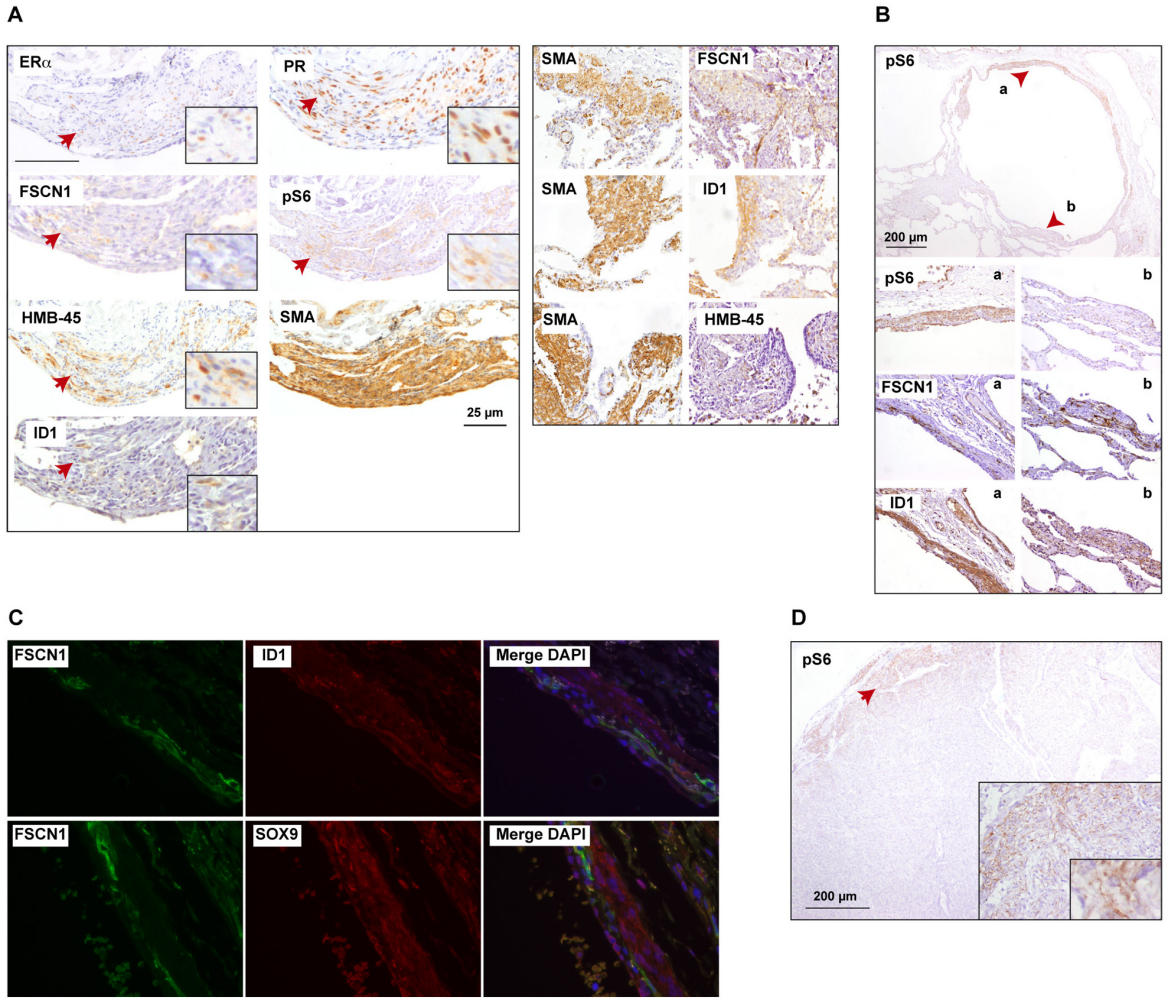
Intra-tissue and inter-case heterogeneity in the staining of canonical and novel LAM biomarkers. (A) Left panels, details of immunohistochemical results for canonical and novel biomarkers (FSNC1 and ID1) in a given LAM lesion, revealing expression heterogeneity. Right panels, evidence of heterogeneity based on the staining of SMA and FSCN1, SMA and ID1, and SMA and HMB-45 in three independent lesions/cases. (B) Top panel, heterogeneity for pS6 staining in a characteristic LAM cystic structure. The arrows mark two different tissue regions (a, b) that are magnified (bottom panels) for the immunohistochemical results of pS6 and the novel biomarkers. (C) Double immunofluorescence staining results also show intra-tissue heterogeneity for the novel biomarkers. (D) In large LAM lesions, pS6 is mostly apparent at the front.

The potential for differential expression correlations and the observed heterogeneity could indicate the existence of diverse cell status and/or phenotypes in LAM lesions. This observation has been raised previously [7,53–55] and can be appreciated in the results for the canonical biomarkers; for example, by comparing the expressions of ERa, PR and SMA, the former being much less representative in a given lesion (Fig 5A). In this regard, pS6 staining also indicated potential intra-tissue heterogeneity for mTORC1 activity (Fig 5B, top panel), which in this case appeared to correlate with the positivity of ID1 but not of FSCN1 (Fig 5B, bottom panels). This complexity was further exposed by double immunostaining assays, which revealed partial co-localization for these biomarkers and SOX9; this observation was more evident between FSCN1 and SOX9, which has an unknown functional significance but could indicate protein co-expression (Fig 5C). Moreover, in large LAM lesions, pS6 staining was principally apparent at the periphery of the lesion (Fig 5D), which would suggest a link to active cell proliferation. Collectively, the data reveal novel biomarkers of LAM that are associated with breast cancer stemness features and further support the existence of relevant heterogeneity among LAM cells and/or lesions.

### Assessment of biomarkers in *Tsc2*-deficient cell models

**Fig 6:**
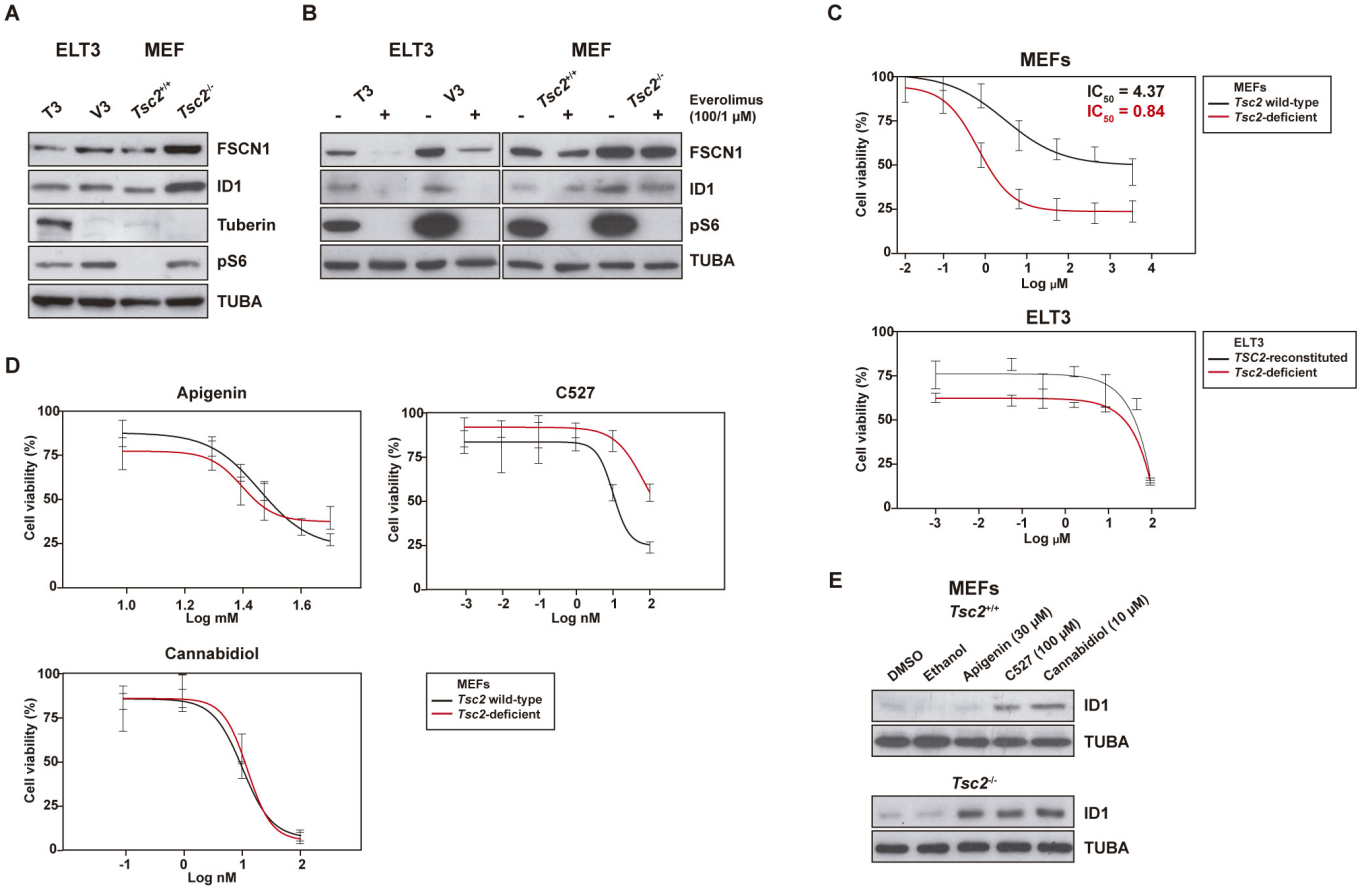
Expression of biomarkers and ID1-based therapeutic evaluation in *Tsc2*-defient cells. (A) Western blot results for FSCN1 and ID1, and controls tuberin, pS6 and TUBA in two *Tsc2*-deficient cell models and their control counterparts. (B) Western blot results for the same biomarkers in cells exposed to everolimus or control solution. (C) Graphs showing the dose-response curves of MEF and ELT3 cell lines exposed to everolimus. (D) Graphs showing the dose-response curves of MEF cell lines exposed to each one of the ID1-expression inhibitors. (E) Western blot results for ID1 and control TUBA in MEF cell lines exposed to the corresponding inhibitors or control solutions.

To further evaluate the relevance of the novel LAM biomarkers, we analyzed their expression level in two cell models of tuberin deficiency: MEFs derived from littermate embryos with the *Tsc2* ^-/-^/*Tp53* ^-/-^ genotype and *Tsc2*-deficient ELT3 (V3) cells. These models were compared to their control counterparts: *Tsc2* ^+/+^/*Tp53* ^-/-^ MEFs and ELT3 reconstituted with human *TSC2* (T3), respectively. Thus, Western blot analyses revealed over-expression of FSCN1 and ID1, particularly in the *Tsc2*-deficient MEFs (Fig 6A). Treatment with everolimus also revealed differences between the models; ELT3 cell lines showed a higher degree of down-regulation of FSCN1 and ID1 with exposure to the rapalog (Fig 6B). Cell type and/or signaling interplay and molecular specificities might explain the differential consequences of mTOR inhibition on the expression of the biomarkers.

Since all LAM lesions revealed ID1 positivity and compounds are available that target the expression or stability of this factor in cancer cells (apigenin, C527, and cannabidiol) [56–59], we next sought to evaluate the selective effect of these compounds in *Tsc2*-deficient cells; particularly in MEFs because this setting revealed a better dose-response relationship for everolimus (Fig 6C). Yet, none of the three ID1 inhibitors revealed higher sensitivity in *Tsc2*-deficient cells (Fig 6D). Surprisingly, cell exposure to the inhibitors led to a relative increase in ID1 expression (Fig 6E). This effect, which is opposite to the effect previously described in cancer cells, might be due to the specificity of the models and/or to interactions with additional targets for those compounds. As novel ID1 inhibitors may be developed, higher specificities may be required for the evaluation in *Tsc2*-deficient cells.

## Discussion

In this study we test the hypothesis that the metastatic properties of LAM cells could be further depicted using knowledge of breast cancer tropism to lung, and identify the expression of metastatic mediators and cancer cell stemness molecular determinants in LAM lesions. The hypothesis was led by the observation that primary breast tumors with relatively low *TSC1/2* expression and predicted enhancement of mTORC1 signaling preferentially metastasize to lung. Consistently, significant co-expression (in the direction of promoting metastasis) was identified between *TSC1/2* and several of the genes that made up a seminal lung metastasis signature. In addition, depletion of *TSC2* in MCF7 breast cancer cells led to a significant gene expression activation of the signature. Collectively, these results are in agreement with previous studies demonstrating an association between low expression of hamartin or tuberin and poor breast cancer clinical outcome [24], between enhanced AKT-mTOR signaling and poor post-relapse breast cancer survival [60], and between depletion of tuberin in mice and breast cancer progression and metastasis [25]. While these observations could indicate a molecular and/or biological link between breast cancer metastatic potential and LAM pathogenesis, the precise role of the identified biomarkers downstream of mTOR signaling remains to be determined. There is growing evidence for the involvement of mTOR in the regulation of stem cell biology [39,61,62], thus the biomarkers might be linked to a down-stream transcriptional program characteristic of this setting.

The function of FSCN1 and ID1 in promoting normal angiogenesis and cancer metastasis is well known [27,37,63]. In addition, the ID proteins are members of a family of basic Helix-Loop-Helix (bHLH) transcription factors whose expression has been shown to be deregulated in different cancer types, being associated with poor-prognosis [63,64]. Over-expression of IDs promotes the maintenance of a stem cell phenotype [40], and the identification of ALDH1, CD61 and SOX9 further endorses the presence of a stem cell-like phenotype in LAM lesions [65]. Importantly, since all LAM lesions revealed ID1 positivity, compounds targeting this factor could potentially exhibit therapeutic benefit in LAM; however, the compounds tested in this study did not show specificity in *Tsc2*-deficient cells or the expected molecular down-regulation [56–59], which would discourage their use in combination with rapamycin or rapalogs. Additional studies may be warranted using specific compounds targeting ID1 function and/or based on the newly defined biomarkers. The potential of using this information to target LAM is further supported by the observation that cyclooxygenase-2, encoded by *PTGS2*, is over-expressed in *TSC2/Tsc2*-deficient cells and constitutes a therapeutic target for LAM [66]; importantly, *PTGS2* is also included in the seminal breast cancer lung metastasis signature [27], being anti-correlated with *TSC1/2* (this study).

The immunohistochemical results also showed inter- and intra-tissue heterogeneity, which further suggests the involvement of LAM cells of different types and/or different proliferation/differentiation status. Although differences in proliferation are likely to exist based on the staining results for pS6 at the front of large LAM lesions, the observed molecular and phenotypic differences could also have been initiated in the tissue of origin and/or through metastatic spread [67]. In this regard, it has been shown that LAM cells proliferate extensively in the lymphatic system and line the thoracic duct, possibly making metastasis to the lung a simple mechanistic phenomenon. Nonetheless, identification in LAM lesions of the expression of specific mediators of lung metastasis could indicate that these molecules particularly facilitate the seeding and/or colonization of the lung. For different cancer types, it has been clearly shown that specificities exist for the preferred tissue/organ of metastasis depending on the molecular profiles of the tumors [68]. The associations observed here for breast cancer metastasis are in agreement with this concept. However, the functional role of the defined biomarkers cannot be clearly demonstrated unless in vivo depletion studies are performed and/or the tissue/organ of origin of LAM cells is defined in each patient. Since the cell origin(s) of LAM is unknown, the expression levels of biomarkers cannot be definitively categorized (relative to the cell origin).

Given the functional relevance of the sub-cellular localization of ID1 and SOX9 [38,45], the differences observed across LAM cases could potentially inform about disease diagnosis and progression. Intriguingly, common genetic variation in *SOX9* has been associated with lung function in the general population [69], which further leads us to speculate that SOX9 function could act as a modifier of LAM pathophysiology. Moreover, *SOX9* expression characterizes alveolar bipotential progenitors and surfactant-secreting cuboidal alveolar type 2 cells [47], which are altered in related lung diseases such as idiopathic pulmonary fibrosis. Additional studies on the cell origin and on related cancer types, such as smooth muscle sarcomas and metastasizing uterine leiomyoma, are also required to evaluate the functional relevance of these observations. Collectively, the results of our study suggest that further understanding of the molecular and cellular basis of breast cancer metastasis to lung may complementarily reveal fundamental insights for LAM.

## Conclusions

Starting from the observation that breast tumors which preferentially metastasize to lung are characterized by low *TSC1/2* expression and possibly by activated mTORC1 signaling, this study reveals five novel biomarkers for LAM lesions: two recognized lung metastasis mediators, FSCN1 and ID1, and three recognized stem and/or progenitor cell regulators, ALDH1, CD61, and SOX9. The examination of these biomarkers further reveals heterogeneity between and within LAM cases. Together, these results might help to further decipher the pathological features and/or tissue origin of LAM cells.

## Supporting Information

S1 Table LAM patients and immunohistochemical results. Clinical data and immunohistochemical results for ALDH1, CD61, FSCN1, ID1, pS6, and SOX9.
(XLSX)

S1 Fig Replication of the association between mTOR pathway gene expression and breast cancer metastasis to lung. (A) Unsupervised clustering of *TSC1/2* (top panels) and *ESR1* (bottom panels) expression profiles across primary breast tumors that produced lung metastases (left panels) and those that produced non-lung metastases (right panels). The data correspond to the GEO GSE11078. All *TSC1/2* probes revealed differential expression between the tumor groups (two-tailed *t*-test *P* values < 0.05). (B) GSEA graphical output for the association analysis between the gene set corresponding to the mTOR pathway and the differential expression between tumors that developed lung versus non-lung metastases. The top panel corresponds to the gene rank according to the *P* value of the *t*-test for differential expression; the bottom panel corresponds to the gene rank according to the *t*-test statistic value. The top genes whose expression differentiate the two tumors types are shown. The GSEA enrichment score and the nominal *P* values are also shown. (C) Kaplan-Meier survival curves based on categorization of *TSC2* expression in tertiles, using GEO GSE2034. The top and bottom panels correspond to ERa-positive and ERa-negative tumors, respectively. The left panels correspond to the analysis of the complete dataset and the right panels correspond to the analysis excluding the 25% of tumors with the lowest (in the ERa-positive set) or the highest (in the ERa-negative set) *ESR1* expression values. The *P* values of the log-rank test when using the complete dataset are shown.
(EPS)

S2 Fig Assessment of osteosarcoma gene expression associations with lung metastasis. Unsupervised hierarchical gene expression clustering of *TSC1/2* and genes that constitute the canonical breast cancer lung metastasis signature, across osteosarcoma tumor samples (top graph shows indications on which tumors produced lung metastasis). The data correspond to GEO GSE14827.
(EPS)

S3 Fig Histopathological and immunohistochemical characterization of LAM lung tissue. (A) Representative hematoxylin-eosin images of lung tissue from a LAM patient. Arrows mark magnified fields shown in the insets. (B) Representative immunohistochemistry results for canonical LAM diagnostic biomarkers.
(EPS)

## References

[1] Blei F Lymphangiomatosis: clinical overview. Lymphat Res Biol. 2011; 9: 185–190. 10.1089/lrb.2011.0020 22196283

[2] Hohman DW, Noghrehkar D, Ratnayake S Lymphangioleiomyomatosis: A review. Eur J Intern Med. 2008; 19: 319–324. 10.1016/j.ejim.2007.10.015 18549932

[3] McCormack FX Lymphangioleiomyomatosis: a clinical update. Chest. 2008; 133: 507–516. 10.1378/chest.07-0898 18252917

[4] Taveira-DaSilva AM, Steagall WK, Moss J Lymphangioleiomyomatosis. Cancer Control. 2006; 13: 276–285. 17075565 10.1177/107327480601300405

[5] Chu SC, Horiba K, Usuki J, Avila NA, Chen CC, Travis WD, et al Comprehensive evaluation of 35 patients with lymphangioleiomyomatosis. Chest. 1999; 115: 1041–1052. 10208206 10.1378/chest.115.4.1041

[6] Matsui K, Takeda K, Yu ZX, Valencia J, Travis WD, Moss J, et al Downregulation of estrogen and progesterone receptors in the abnormal smooth muscle cells in pulmonary lymphangioleiomyomatosis following therapy. An immunohistochemical study. Am J Respir Crit Care Med. 2000; 161: 1002–1009. 10712355 10.1164/ajrccm.161.3.9904009

[7] Gao L, Yue MM, Davis J, Hyjek E, Schuger L In pulmonary lymphangioleiomyomatosis expression of progesterone receptor is frequently higher than that of estrogen receptor. Virchows Arch. 2014; 464: 495–503. 10.1007/s00428-014-1559-9 24570392

[8] Yu J, Henske EP mTOR activation, lymphangiogenesis, and estrogen-mediated cell survival: the "perfect storm" of pro-metastatic factors in LAM pathogenesis. Lymphat Res Biol. 2010; 8: 43–49. 10.1089/lrb.2009.0020 20235886 PMC2883473

[9] McCormack FX, Travis WD, Colby TV, Henske EP, Moss J Lymphangioleiomyomatosis: calling it what it is: a low-grade, destructive, metastasizing neoplasm. Am J Respir Crit Care Med. 2012; 186: 1210–1212. 10.1164/rccm.201205-0848OE 23250499 PMC3622443

[10] Muzykewicz DA, Sharma A, Muse V, Numis AL, Rajagopal J, Thiele EA *TSC1* and *TSC2* mutations in patients with lymphangioleiomyomatosis and tuberous sclerosis complex. J Med Genet. 2009; 46: 465–468. 10.1136/jmg.2008.065342 19419980

[11] Sato T, Seyama K, Fujii H, Maruyama H, Setoguchi Y, Iwakami S, et al Mutation analysis of the *TSC1* and *TSC2* genes in Japanese patients with pulmonary lymphangioleiomyomatosis. J Hum Genet. 2002; 47: 20–28. 11829138 10.1007/s10038-002-8651-8

[12] Carsillo T, Astrinidis A, Henske EP Mutations in the tuberous sclerosis complex gene *TSC2* are a cause of sporadic pulmonary lymphangioleiomyomatosis. Proc Natl Acad Sci U S A. 2000; 97: 6085–6090. 10823953 10.1073/pnas.97.11.6085 PMC18562

[13] Astrinidis A, Khare L, Carsillo T, Smolarek T, Au KS, Northrup H, et al Mutational analysis of the tuberous sclerosis gene *TSC2* in patients with pulmonary lymphangioleiomyomatosis. J Med Genet. 2000; 37: 55–57. 10633137 10.1136/jmg.37.1.55 PMC1734439

[14] Crino PB, Nathanson KL, Henske EP The tuberous sclerosis complex. N Engl J Med. 2006; 355: 1345–1356. 17005952 10.1056/NEJMra055323

[15] Zoncu R, Efeyan A, Sabatini DM mTOR: from growth signal integration to cancer, diabetes and ageing. Nat Rev Mol Cell Biol. 2011; 12: 21–35. 10.1038/nrm3025 21157483 PMC3390257

[16] McCormack FX, Inoue Y, Moss J, Singer LG, Strange C, Nakata K, et al Efficacy and safety of sirolimus in lymphangioleiomyomatosis. N Engl J Med. 2011; 364: 1595–1606. 10.1056/NEJMoa1100391 21410393 PMC3118601

[17] Taveira-DaSilva AM, Pacheco-Rodriguez G, Moss J The natural history of lymphangioleiomyomatosis: markers of severity, rate of progression and prognosis. Lymphat Res Biol. 2010; 8: 9–19. 10.1089/lrb.2009.0024 20235883 PMC2883494

[18] Crooks DM, Pacheco-Rodriguez G, DeCastro RM, McCoy JP Jr., Wang JA, Kumaki F, et al Molecular and genetic analysis of disseminated neoplastic cells in lymphangioleiomyomatosis. Proc Natl Acad Sci U S A. 2004; 101: 17462–17467. 15583138 10.1073/pnas.0407971101 PMC536045

[19] Seyama K, Kumasaka T, Kurihara M, Mitani K, Sato T Lymphangioleiomyomatosis: a disease involving the lymphatic system. Lymphat Res Biol. 2010; 8: 21–31. 10.1089/lrb.2009.0018 20235884

[20] Karbowniczek M, Astrinidis A, Balsara BR, Testa JR, Lium JH, Colby TV, et al Recurrent lymphangiomyomatosis after transplantation: genetic analyses reveal a metastatic mechanism. Am J Respir Crit Care Med. 2003; 167: 976–982. 12411287 10.1164/rccm.200208-969OC

[21] Bittmann I, Rolf B, Amann G, Lohrs U Recurrence of lymphangioleiomyomatosis after single lung transplantation: new insights into pathogenesis. Hum Pathol. 2003; 34: 95–98. 12605373 10.1053/hupa.2003.50

[22] Badri KR, Gao L, Hyjek E, Schuger N, Schuger L, Qin W, et al Exonic mutations of *TSC2/TSC1* are common but not seen in all sporadic pulmonary lymphangioleiomyomatosis. Am J Respir Crit Care Med. 2013; 187: 663–665. 23504366 10.1164/ajrccm.187.6.663 PMC3733437

[23] Hsieh AC, Liu Y, Edlind MP, Ingolia NT, Janes MR, Sher A, et al The translational landscape of mTOR signalling steers cancer initiation and metastasis. Nature. 2012; 485: 55–61. 10.1038/nature10912 22367541 PMC3663483

[24] Jiang WG, Sampson J, Martin TA, Lee-Jones L, Watkins G, Douglas-Jones A, et al Tuberin and hamartin are aberrantly expressed and linked to clinical outcome in human breast cancer: the role of promoter methylation of *TSC* genes. Eur J Cancer. 2005; 41: 1628–1636. 15951164 10.1016/j.ejca.2005.03.023

[25] Nasr Z, Robert F, Porco JA Jr., Muller WJ, Pelletier J eIF4F suppression in breast cancer affects maintenance and progression. Oncogene. 2013; 32: 861–871. 10.1038/onc.2012.105 22484424 PMC4863948

[26] Pacheco-Rodriguez G, Moss J The role of chemokines in migration of metastatic-like lymphangioleiomyomatosis cells. Crit Rev Immunol. 2010; 30: 387–394. 20666708 10.1615/critrevimmunol.v30.i4.40 PMC3021991

[27] Minn AJ, Gupta GP, Siegel PM, Bos PD, Shu W, Giri DD, et al Genes that mediate breast cancer metastasis to lung. Nature. 2005; 436: 518–524. 16049480 10.1038/nature03799 PMC1283098

[28] Landemaine T, Jackson A, Bellahcene A, Rucci N, Sin S, Abad BM, et al A six-gene signature predicting breast cancer lung metastasis. Cancer Res. 2008; 68: 6092–6099. 10.1158/0008-5472.CAN-08-0436 18676831

[29] Wang Y, Klijn JG, Zhang Y, Sieuwerts AM, Look MP, Yang F, et al Gene-expression profiles to predict distant metastasis of lymph-node-negative primary breast cancer. Lancet. 2005; 365: 671–679. 15721472 10.1016/S0140-6736(05)17947-1

[30] Kobayashi E, Masuda M, Nakayama R, Ichikawa H, Satow R, Shitashige M, et al Reduced argininosuccinate synthetase is a predictive biomarker for the development of pulmonary metastasis in patients with osteosarcoma. Mol Cancer Ther. 2010; 9: 535–544. 10.1158/1535-7163.MCT-09-0774 20159990

[31] Subramanian A, Tamayo P, Mootha VK, Mukherjee S, Ebert BL, Gillette MA, et al Gene set enrichment analysis: a knowledge-based approach for interpreting genome-wide expression profiles. Proc Natl Acad Sci U S A. 2005; 102: 15545–15550. 16199517 10.1073/pnas.0506580102 PMC1239896

[32] Kanehisa M, Goto S, Sato Y, Furumichi M, Tanabe M KEGG for integration and interpretation of large-scale molecular data sets. Nucleic Acids Res. 2012; 40: D109–114. 10.1093/nar/gkr988 22080510 PMC3245020

[33] Ferrer I, Mohan P, Chen H, Castellsague J, Gomez-Baldo L, Carmona M, et al Tubers from patients with tuberous sclerosis complex are characterized by changes in microtubule biology through ROCK2 signalling. J Pathol. 2014; 233: 247–257. 10.1002/path.4343 24604753

[34] Gomez-Baldo L, Schmidt S, Maxwell CA, Bonifaci N, Gabaldon T, Vidalain PO, et al TACC3-TSC2 maintains nuclear envelope structure and controls cell division. Cell Cycle. 2010; 9: 1143–1155. 20237422 10.4161/cc.9.6.11018

[35] Anton E, Casanova A, Xaubet A, Roman A, Villena V, Montero MC, et al Lymphangioleiomyomatosis: a study of 72 patients from the Spanish registry. Sarcoidosis Vasc Diffuse Lung Dis. 2009; 26: 85–91. 20560288

[36] Smid M, Wang Y, Zhang Y, Sieuwerts AM, Yu J, Klijn JG, et al Subtypes of breast cancer show preferential site of relapse. Cancer Res. 2008; 68: 3108–3114. 10.1158/0008-5472.CAN-07-5644 18451135

[37] Hashimoto Y, Kim DJ, Adams JC The roles of fascins in health and disease. J Pathol. 2011; 224: 289–300. 10.1002/path.2894 21618240

[38] Nishiyama K, Takaji K, Uchijima Y, Kurihara Y, Asano T, Yoshimura M, et al Protein kinase A-regulated nucleocytoplasmic shuttling of Id1 during angiogenesis. J Biol Chem. 2007; 282: 17200–17209. 17412691 10.1074/jbc.M611609200

[39] Gan B, Sahin E, Jiang S, Sanchez-Aguilera A, Scott KL, Chin L, et al mTORC1-dependent and -independent regulation of stem cell renewal, differentiation, and mobilization. Proc Natl Acad Sci U S A. 2008; 105: 19384–19389. 10.1073/pnas.0810584105 19052232 PMC2593615

[40] Ying QL, Nichols J, Chambers I, Smith A BMP induction of Id proteins suppresses differentiation and sustains embryonic stem cell self-renewal in collaboration with STAT3. Cell. 2003; 115: 281–292. 14636556 10.1016/s0092-8674(03)00847-x

[41] Romero-Lanman EE, Pavlovic S, Amlani B, Chin Y, Benezra R Id1 maintains embryonic stem cell self-renewal by up-regulation of Nanog and repression of Brachyury expression. Stem Cells Dev. 2012; 21: 384–393. 10.1089/scd.2011.0428 22013995

[42] Ginestier C, Hur MH, Charafe-Jauffret E, Monville F, Dutcher J, Brown M, et al ALDH1 is a marker of normal and malignant human mammary stem cells and a predictor of poor clinical outcome. Cell Stem Cell. 2007; 1: 555–567. 10.1016/j.stem.2007.08.014 18371393 PMC2423808

[43] Lo PK, Kanojia D, Liu X, Singh UP, Berger FG, Wang Q, et al CD49f and CD61 identify Her2/neu-induced mammary tumor-initiating cells that are potentially derived from luminal progenitors and maintained by the integrin-TGFbeta signaling. Oncogene. 2012; 31: 2614–2626. 10.1038/onc.2011.439 21996747 PMC3260386

[44] Fu N, Lindeman GJ, Visvader JE The mammary stem cell hierarchy. Curr Top Dev Biol. 2014; 107: 133–160. 10.1016/B978-0-12-416022-4.00005-6 24439805

[45] Chakravarty G, Moroz K, Makridakis NM, Lloyd SA, Galvez SE, Canavello PR, et al Prognostic significance of cytoplasmic SOX9 in invasive ductal carcinoma and metastatic breast cancer. Exp Biol Med (Maywood). 2011; 236: 145–155. 21321311 10.1258/ebm.2010.010086

[46] Guo W, Keckesova Z, Donaher JL, Shibue T, Tischler V, Reinhardt F, et al Slug and Sox9 cooperatively determine the mammary stem cell state. Cell. 2012; 148: 1015–1028. 10.1016/j.cell.2012.02.008 22385965 PMC3305806

[47] Treutlein B, Brownfield DG, Wu AR, Neff NF, Mantalas GL, Espinoza FH, et al Reconstructing lineage hierarchies of the distal lung epithelium using single-cell RNA-seq. Nature. 2014; 509: 371–375. 10.1038/nature13173 24739965 PMC4145853

[48] Honeth G, Schiavinotto T, Vaggi F, Marlow R, Kanno T, Shinomiya I, et al Models of breast morphogenesis based on localization of stem cells in the developing mammary lobule. Stem Cell Reports. 2015; 4: 699–711. 10.1016/j.stemcr.2015.02.013 25818813 PMC4400614

[49] Boecker W, Moll R, Dervan P, Buerger H, Poremba C, Diallo RI, et al Usual ductal hyperplasia of the breast is a committed stem (progenitor) cell lesion distinct from atypical ductal hyperplasia and ductal carcinoma in situ. J Pathol. 2002; 198: 458–467. 12434415 10.1002/path.1241

[50] Lin HJ, Hsieh FC, Song H, Lin J Elevated phosphorylation and activation of PDK-1/AKT pathway in human breast cancer. Br J Cancer. 2005; 93: 1372–1381. 16288304 10.1038/sj.bjc.6602862 PMC2361529

[51] Wagner SN, Wagner C, Schultewolter T, Goos M Analysis of Pmel17/gp100 expression in primary human tissue specimens: implications for melanoma immuno- and gene-therapy. Cancer Immunol Immunother. 1997; 44: 239–247. 9222283 10.1007/s002620050379 PMC11037831

[52] Bonetti F, Colombari R, Manfrin E, Zamboni G, Martignoni G, Mombello A, et al Breast carcinoma with positive results for melanoma marker (HMB-45). HMB-45 immunoreactivity in normal and neoplastic breast. Am J Clin Pathol. 1989; 92: 491–495. 2552794 10.1093/ajcp/92.4.491

[53] Steagall WK, Zhang L, Cai X, Pacheco-Rodriguez G, Moss J Genetic heterogeneity of circulating cells from patients with lymphangioleiomyomatosis with and without lung transplantation. Am J Respir Crit Care Med. 2015; 191: 854–856. 10.1164/rccm.201412-2170LE 25830522 PMC4407489

[54] Cai X, Pacheco-Rodriguez G, Fan QY, Haughey M, Samsel L, El-Chemaly S, et al Phenotypic characterization of disseminated cells with *TSC2* loss of heterozygosity in patients with lymphangioleiomyomatosis. Am J Respir Crit Care Med. 2010; 182: 1410–1418. 10.1164/rccm.201003-0489OC 20639436 PMC3029931

[55] Delaney SP, Julian LM, Stanford WL The neural crest lineage as a driver of disease heterogeneity in Tuberous Sclerosis Complex and Lymphangioleiomyomatosis. Front Cell Dev Biol. 2014; 2: 69 10.3389/fcell.2014.00069 25505789 PMC4243694

[56] McAllister SD, Murase R, Christian RT, Lau D, Zielinski AJ, Allison J, et al Pathways mediating the effects of cannabidiol on the reduction of breast cancer cell proliferation, invasion, and metastasis. Breast Cancer Res Treat. 2011; 129: 37–47. 10.1007/s10549-010-1177-4 20859676 PMC3410650

[57] Li ZD, Hu XW, Wang YT, Fang J Apigenin inhibits proliferation of ovarian cancer A2780 cells through Id1. FEBS Lett. 2009; 583: 1999–2003. 10.1016/j.febslet.2009.05.013 19447105

[58] Mistry H, Hsieh G, Buhrlage SJ, Huang M, Park E, Cuny GD, et al Small-molecule inhibitors of USP1 target ID1 degradation in leukemic cells. Mol Cancer Ther. 2013; 12: 2651–2662. 10.1158/1535-7163.MCT-13-0103-T 24130053 PMC4089878

[59] Soroceanu L, Murase R, Limbad C, Singer E, Allison J, Adrados I, et al Id-1 is a key transcriptional regulator of glioblastoma aggressiveness and a novel therapeutic target. Cancer Res. 2013; 73: 1559–1569. 10.1158/0008-5472.CAN-12-1943 23243024 PMC3594064

[60] Tobin NP, Harrell JC, Lovrot J, Egyhazi Brage S, Frostvik Stolt M, Carlsson L, et al Molecular subtype and tumor characteristics of breast cancer metastases as assessed by gene expression significantly influence patient post-relapse survival. Ann Oncol. 2015; 26: 81–88. 10.1093/annonc/mdu498 25361981 PMC4269343

[61] Russell RC, Fang C, Guan KL An emerging role for TOR signaling in mammalian tissue and stem cell physiology. Development. 2011; 138: 3343–3356. 10.1242/dev.058230 21791526 PMC3143559

[62] Zhou J, Wulfkuhle J, Zhang H, Gu P, Yang Y, Deng J, et al Activation of the PTEN/mTOR/STAT3 pathway in breast cancer stem-like cells is required for viability and maintenance. Proc Natl Acad Sci U S A. 2007; 104: 16158–16163. 17911267 10.1073/pnas.0702596104 PMC2042178

[63] Perk J, Iavarone A, Benezra R Id family of helix-loop-helix proteins in cancer. Nat Rev Cancer. 2005; 5: 603–614. 16034366 10.1038/nrc1673

[64] Hasskarl J, Munger K Id proteins—tumor markers or oncogenes? Cancer Biol Ther. 2002; 1: 91–96. 12170780 10.4161/cbt.50

[65] Finlay G The LAM cell: what is it, where does it come from, and why does it grow? Am J Physiol Lung Cell Mol Physiol. 2004; 286: L690–693. 15003933 10.1152/ajplung.00311.2003

[66] Li C, Lee PS, Sun Y, Gu X, Zhang E, Guo Y, et al Estradiol and mTORC2 cooperate to enhance prostaglandin biosynthesis and tumorigenesis in *TSC2*-deficient LAM cells. J Exp Med. 2014; 211: 15–28. 10.1084/jem.20131080 24395886 PMC3892971

[67] Mani SA, Guo W, Liao MJ, Eaton EN, Ayyanan A, Zhou AY, et al The epithelial-mesenchymal transition generates cells with properties of stem cells. Cell. 2008; 133: 704–715. 10.1016/j.cell.2008.03.027 18485877 PMC2728032

[68] Valastyan S, Weinberg RA Tumor metastasis: molecular insights and evolving paradigms. Cell. 2011; 147: 275–292. 10.1016/j.cell.2011.09.024 22000009 PMC3261217

[69] Hancock DB, Artigas MS, Gharib SA, Henry A, Manichaikul A, Ramasamy A, et al Genome-wide joint meta-analysis of SNP and SNP-by-smoking interaction identifies novel loci for pulmonary function. PLoS Genet. 2012; 8: e1003098 10.1371/journal.pgen.1003098 23284291 PMC3527213

[70] TCGA Comprehensive molecular portraits of human breast tumours. Nature. 2012; 490: 61–70. 10.1038/nature11412 23000897 PMC3465532

