# Correction: Lymphangioleiomyomatosis Biomarkers Linked to Lung Metastatic Potential and Cell Stemness

**DOI:** 10.1371/journal.pone.0207586

**Published:** 2018-11-12

**Authors:** Gorka Ruiz de Garibay, Carmen Herranz, Alicia Llorente, Jacopo Boni, Jordi Serra-Musach, Francesca Mateo, Helena Aguilar, Laia Gómez-Baldó, Anna Petit, August Vidal, Fina Climent, Javier Hernández-Losa, Álex Cordero, Eva González-Suárez, José Vicente Sánchez-Mut, Manel Esteller, Roger Llatjós, Mar Varela, José Ignacio López, Nadia García, Ana I. Extremera, Anna Gumà, Raúl Ortega, María Jesús Plà, Adela Fernández, Sònia Pernas, Catalina Falo, Idoia Morilla, Miriam Campos, Miguel Gil, Antonio Román, María Molina-Molina, Piedad Ussetti, Rosalía Laporta, Claudia Valenzuela, Julio Ancochea, Antoni Xaubet, Álvaro Casanova, Miguel Angel Pujana

The following information is missing from the Funding section: This study was supported by the Spanish Ministry of Science and Innovation, European Regional Development Fund (FEDER).
